# Supplementary material for: Single-cell profiling reveals distinct adaptive immune hallmarks in MDA5+ dermatomyositis with therapeutic implications
Source: Nat Commun. 2022 Oct 29;13:6458. doi: 10.1038/s41467-022-34145-4 (PMC9617246; doi:10.1038/s41467-022-34145-4)
Supplement: Supplementary file 1 — Supplementary Information [file 41467_2022_34145_MOESM1_ESM.pdf]

Supplementary Information

Single-cell Profiling Reveals Distinct Adaptive Immune Hallmarks in MDA5$^+$ Dermatomyositis with Therapeutic Implications

Yan Ye, Zechuan Chen, Shan Jiang, Fengyun Jia, Teng Li, Xia Lu, Jing Xue, Xinyue Lian, Jiaqiang Ma, Pei Hao, Liangjing Lu, Shuang Ye, Nan Shen, Chunde Bao, Qiong Fu*, Xiaoming Zhang*

*Corresponding authors: xmzhang@ips.ac.cn (X.M.Z.), fuqiong@renji.com (Q.F.)

Supplementary materials:

Supplementary Figures 1-7

Supplementary Tables 1-6

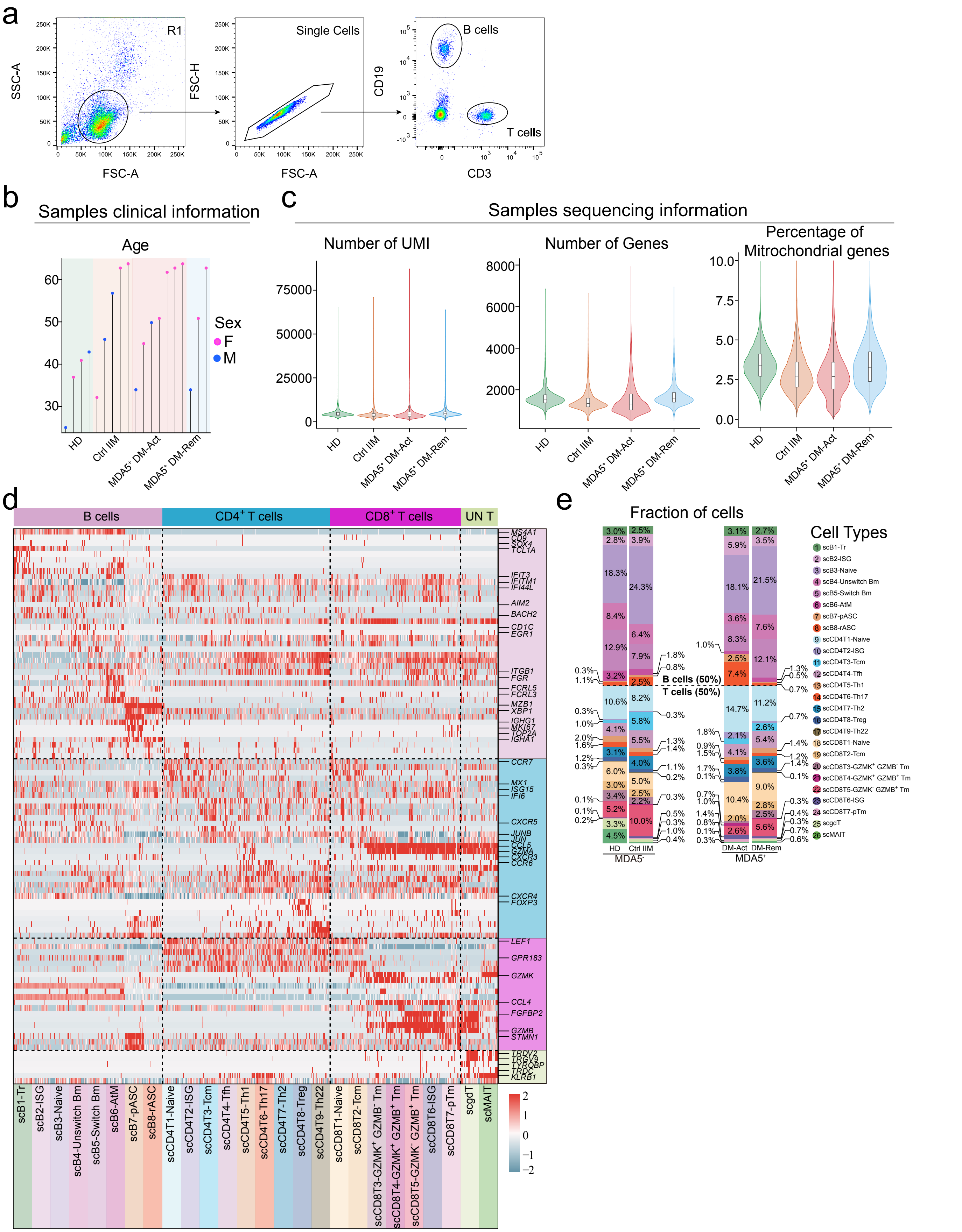

Supplementary Figure 1: Single-cell atlas of peripheral B and T cells from MDA5+ DM patients and controls. a. Flow cytometry gating strategy to sort peripheral B and T cells. **b.** The age and sex distributions of all samples used for peripheral B/T cell sequencing. The points are colored by sex and the background region is colored by group. **c.** Violin plots showing the distributions of unique molecular identifier (UMI) counts per cell, gene counts per cell and percentage of mitochondrial transcripts per cell from HD (n=23,652 cells), Ctrl IIM (n=13,846 cells), MDA5+ DM-Act (n=56,085 cells) and MDA5+ DM-Rem (n=17,617 cells) groups. Box plot center, box and whiskers correspond to median, IQR and 1.5 × IQR, respectively. **d.** Heatmap showing top5 marker genes for each cluster. **e.** Stacked column charts showing the proportions of indicated clusters in B cell compartment (total B cells as 50%) and T cell compartment (total T cells as 50%) across four groups. Clusters are color-coded.

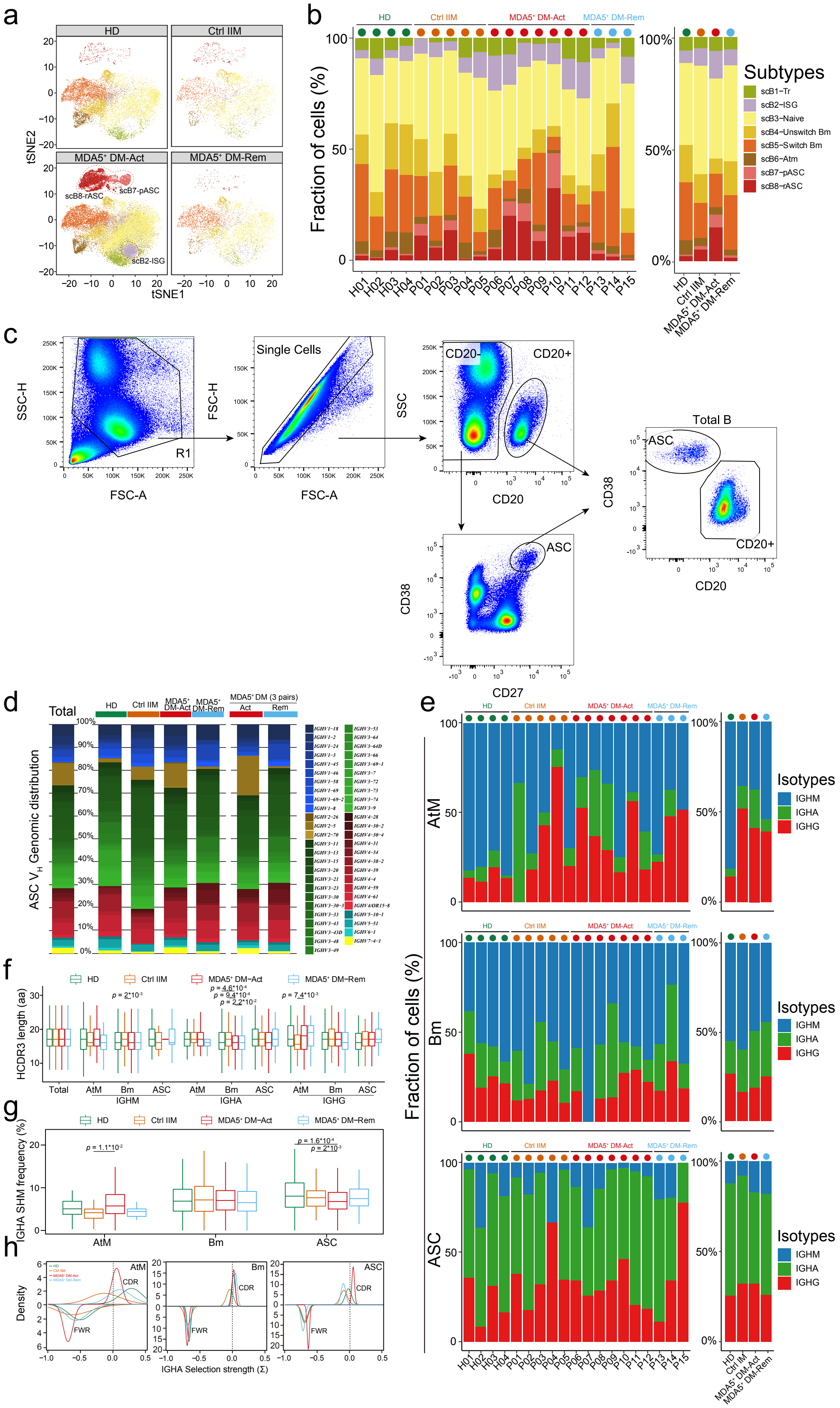

Supplementary Figure 2: Transcriptome and BCR features of peripheral B cells from MDA5+ DM patients and controls. a. t-SNE plots showing B cell clusters across four groups, color-coded according to cell clusters. **b.** Stacked column charts showing the proportions of B cell clusters from individual donors and indicated groups. **c.** Flow cytometry gating strategy to determine CD20+ B cells and CD20+CD27hiCD38hi antibody-secreting cells (ASCs). **d.** Stacked column charts showing the frequencies of IGHV gene usages of ASCs across four groups. **e.** Stacked column charts showing the frequencies of IGH isotypes (IGHM, IGHA and IGHG) in atypical memory B cells (AtM), memory B cells (Bm) and ASCs across four groups. **f.** Box plots showing the lengths of the immuno-globulin heavy-chain complementarity-determining region 3 (HCDR3) of indicated IGH isotypes of the Atm (IGHM, n=818; IGHA, n=150; IGHG, n=425), Bm (IGHM, n=5,215; IGHA, n=2,659; IGHG, n=2,512) and ASC (IGHM, n=965; IGHA, n=3,176; IGHG, n=1,477) cells. aa: amino acid. **g.** Boxplots showing the somatic hypermutation (SHM) frequencies of IGHA of the Atm (n=150), Bm(n=2,659) and ASC(n=4,008) cells. **h.** Density plots showing the IGHA selection strengths on complementary-determining region (CDR, Upper) and framework region (FWR, Lower) of indicated B cell clusters across four groups. Box plot center, box and whiskers correspond to median, IQR and 1.5 × IQR, respectively, and statistical significance is calculated by two-tailed Mann-Whitney test (f, g).

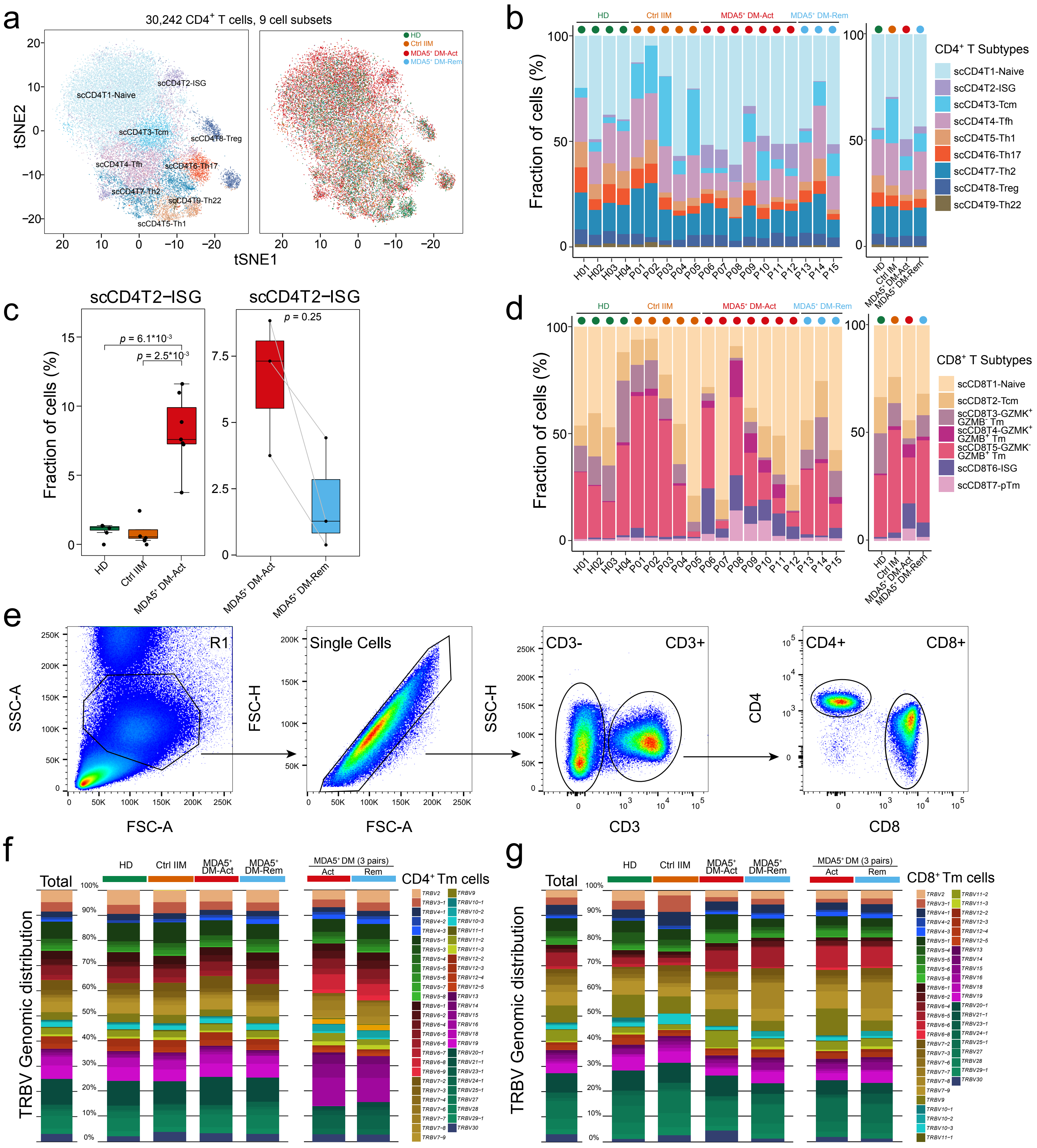

Supplementary Figure 3: Transcriptome and TCR features of peripheral CD4+ and CD8+ T cells from MDA5+ DM patients and controls. a. t-SNE plots showing 9 CD4+ T cell clusters, color-coded according to cell subset (Left) or group (Right). **b.** Stacked column charts showing the proportions of CD4+ T cell clusters from individual donors and indicated groups. **c.** Box plots showing the proportions of scCD4T2-ISG of HD (n=4), Ctrl IIM (n=5), MDA5+ DM-Act (n=7) and MDA5+ DM-Rem(n=3). Box plot center, box and whiskers correspond to median, IQR and 1.5 × IQR, respectively. Statistical significance is calculated by the two-tailed Mann–Whitney test. **d.** Flow cytometry gating strategy to determine CD4+ and CD8+ T cells. **e.** Stacked column charts showing the proportions of CD8+ T cell clusters from individual donors and indicated groups. **f** and **g.** Stacked column charts showing the frequencies of TRBV gene usages of CD4+ Tm (f) or CD8+ Tm (g) across four groups.

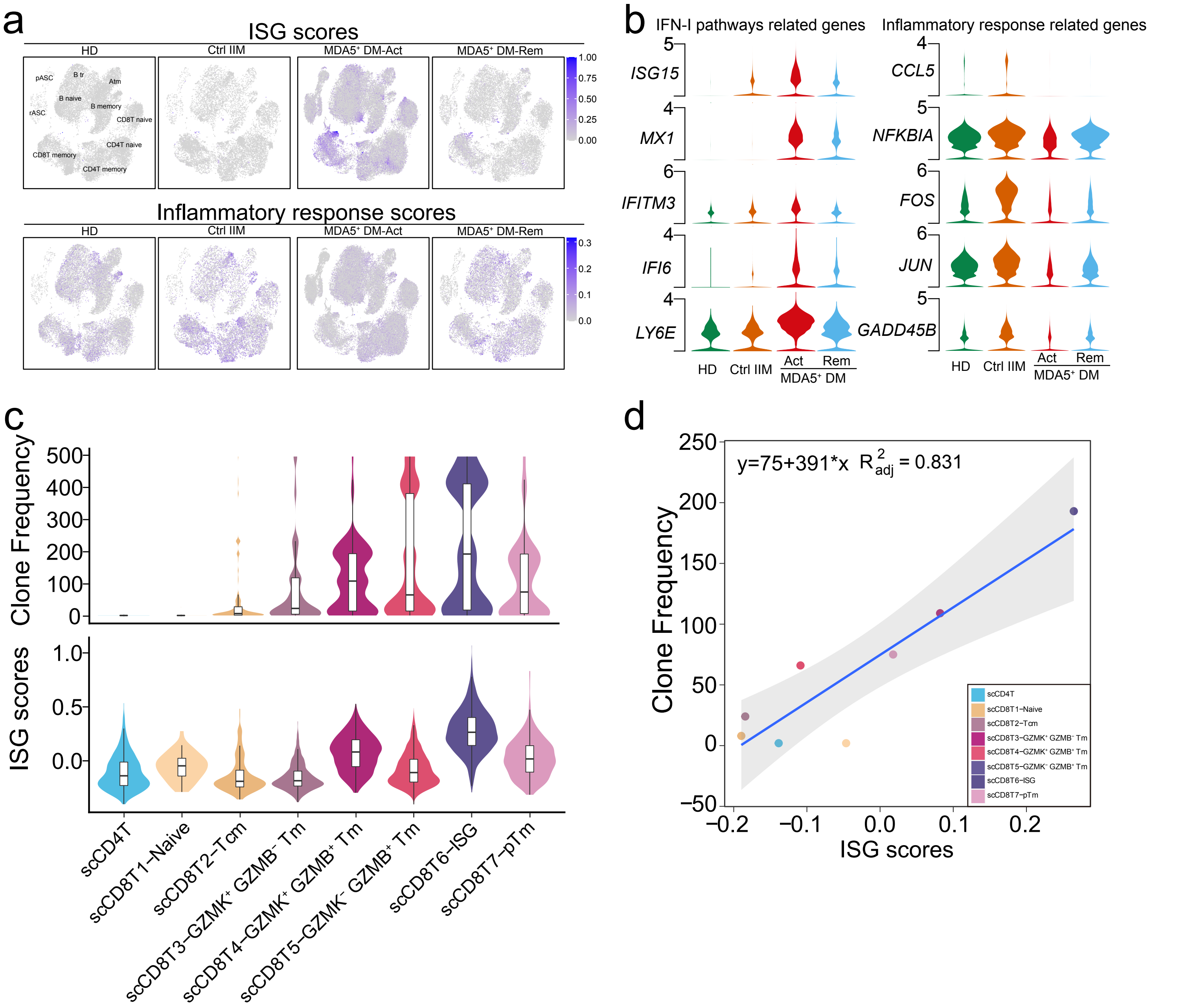

Supplementary Figure 4: Type I IFN and inflammatory pathway analyses of peripheral B and T cells in MDA5+ DM patients. **a.** t-SNE plots showing the projections of ISG scores (Upper) and inflammatory response scores (Lower) on peripheral B and T cells across four groups. **b.** Violin plots showing selected IFN-I pathway related genes (Left) and Inflammatory response related genes (Right) expressed in peripheral B and T cells across four groups. **c.** Violin plots showing the clone frequency (Upper) and ISG scores (Lower) of T cell cluster from the MDA5+ DM-Act group (n= 446 cells; 43 cells; 234 cells; 953 cells; 497 cells; 4,224 cells; 1,067 cells; 379 cells; from left to right). Box plot center, box and whiskers correspond to median, IQR and 1.5 × IQR, respectively. **d.** Correlation analysis between the clone frequency and ISG scores of T cell clusters from the MDA5+ DM-Act group. Spearman’s correlation analysis is applied with a 95% confidence interval.

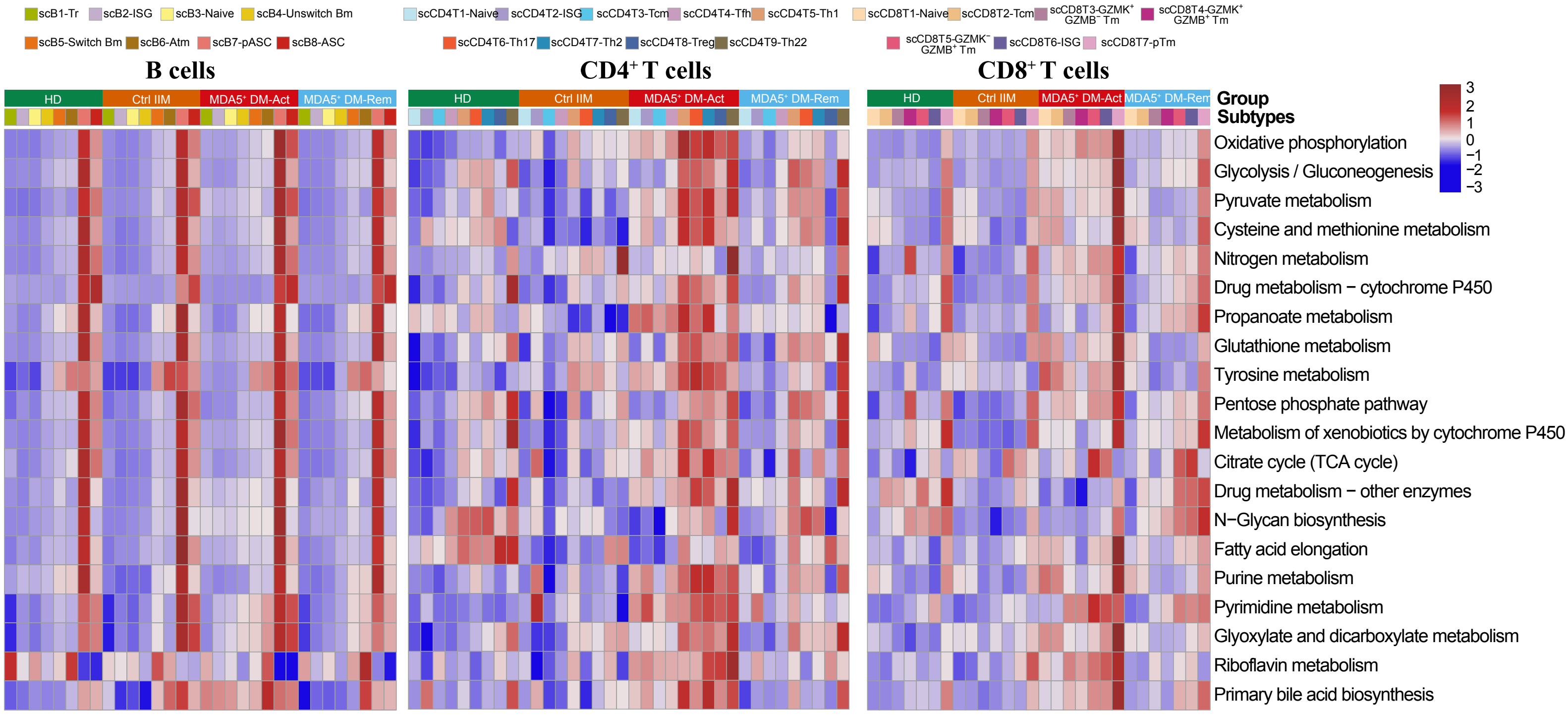

Supplementary Figure 5: Metabolic pathway analysis of B and T cell subsets in MDA5+ DM patients and controls. Heatmap showing the top20 metabolic pathways enriched in the MDA5+ DM-Act group across B cell, CD4+ and CD8+ T cell clusters from four groups. Scale bar represents the scaled ssGSEA scores.

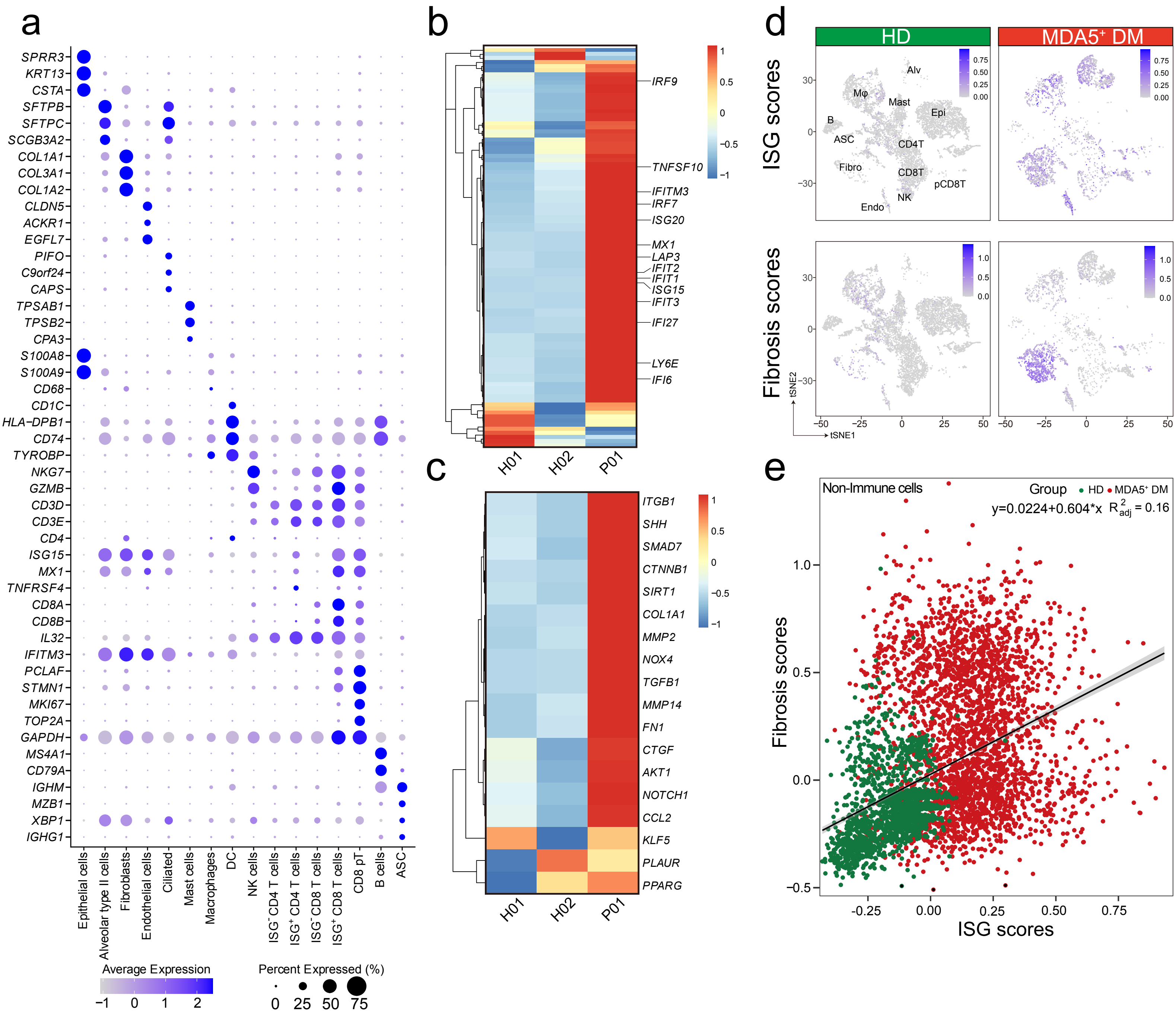

Supplementary Figure 6: Single-cell transcriptome analysis of the lung tissues from one MDA5+ DM patient and two HDs.
a. Bubble plot showing the top5 cell cluster-specific marker genes in lung tissues, with color representing the scaled average expression and sized by the fraction of cells (average expression >0). **b** and **c.** Heatmaps showing the average expressions of signature genes of type-I interferon pathway (b) and fibrosis pathway (c) in individual lung samples. **d.** t-SNE plots showing the projections of ISG scores (Upper) and Fibrosis scores (Lower) on the lung cells from two HDs and one MDA5+ DM patient. **e.** Correlation analysis between the Fibrosis scores and ISG scores of the non-immune cells from two HDs and one MDA5+ DM patient. Spearman's correlation analysis is applied.

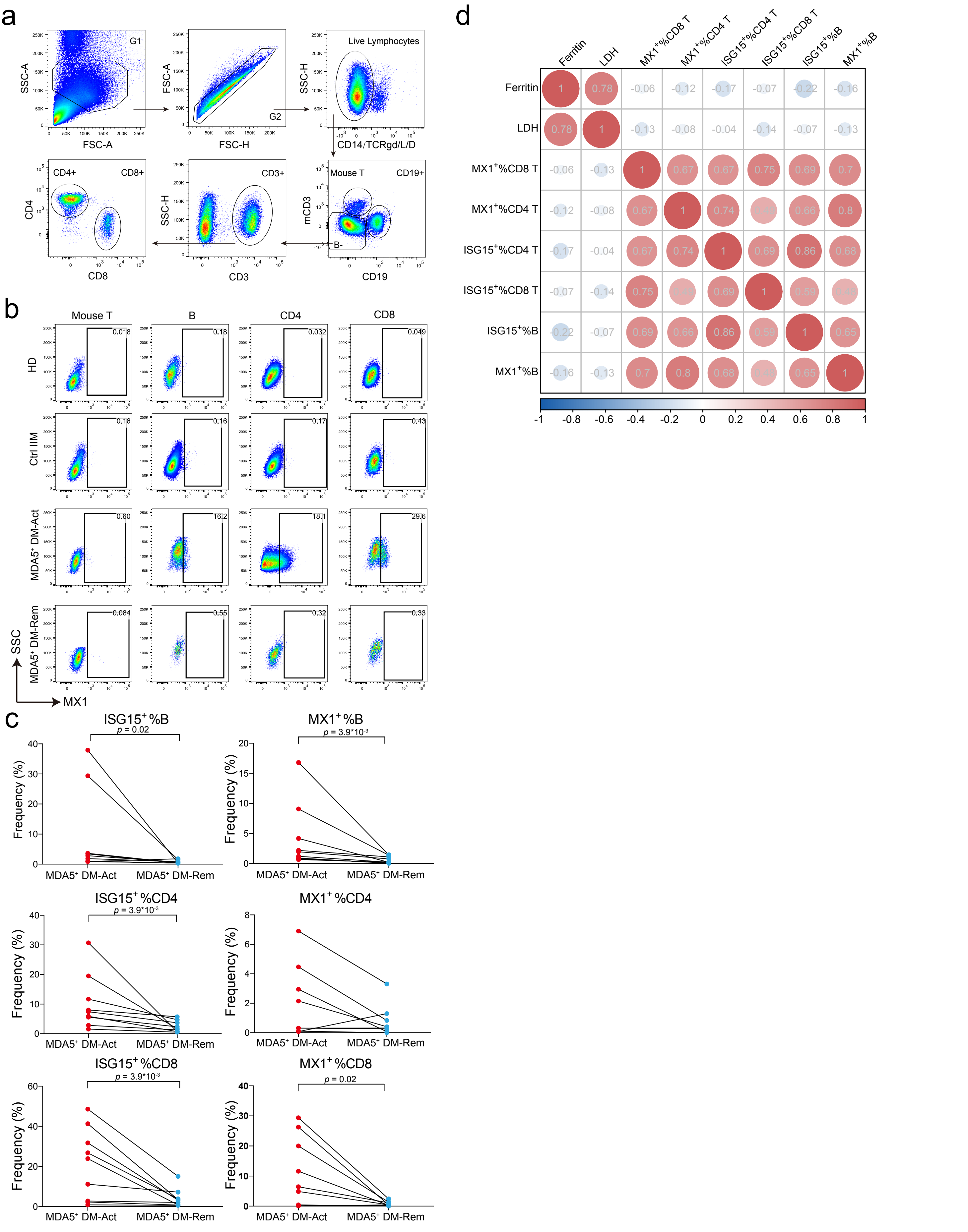

Supplementary Figure 7: Prognostic value of ISG15+CD8+ T cells. a. Flow cytometry gating strategies to determine mouse CD3+ T cells, human CD19+ B cell, CD3+CD4+ and CD3+CD8+ T cells. **b.** Representative flow cytometric plots showing the gating strategies for MX1+ B cell, CD4+ and CD8+ T cells. Mouse CD3+ T cells were used as the negative controls. **c.** Scatter plots showing the frequencies of ISG15+ and MX1+ B cell, CD4+ and CD8+ T cells between active and remitted MDA5+ DM patients (n=9). Statistical significance is calculated by the two-tailed Wilcoxon matched-pairs signed rank test. **d.** Bubble plot showing the correlation coefficients and for the association between indicated variables. Spearman's correlation analysis is applied.

Supplementary Table 1. Clinical information of recruited donors for scRNA-seq

Sample ID	Sex/ Age (year)	Group	Disease Activity/ MITAX	Disease Duration (month)	MSA	CK (U/L)	Ferritin (ng/mL)	LDH (U/L)	Rash	Weaknes s	ILD	Treatment	Outcome
P01	F/63	Ctrl IIM	Active/0.35	12	Jo-1+	212	447.9	372	No	Yes	Yes	Pred	Alive
P02	M/57	Ctrl IIM	Active/0.37	3	PL-7+++	492	381.2	417	No	Yes	Yes	MP, Aza	Alive
P03	F/64	Ctrl IIM	Active/0.33	4	Jo-1+++	281	43.7	307	No	Yes	Yes	Pred,TAC	Alive
P04	M/71	Ctrl IIM	Active/0.33	3	Mi-2++	1223	897.6	567	Yes	Yes	No	Pred,MTX	Alive
P05	F/23	Ctrl IIM	Active/0.57	9	Mi-2++	599	104.2	461	Yes	Yes	No	MP, TAC	Alive
P06	M/51	MDA5+DM-Act	Active/0.43	3	MDA5+	217	1641	283	Yes	No	Yes	MP	Alive
P07[#]	M/34	MDA5+DM-Act	Active/0.43	1	MDA5+++	49	681	229	Yes	No	Yes	MP	Alive
P08[##]	F/63	MDA5+DM-Act	Active/0.48	2	MDA5+++	61	648.6	368	Yes	No	Yes	MP, CTX	Alive
P09	F/64	MDA5+DM-Act	Active/0.33	1	MDA5++	212	762.6	411	Yes	Yes	Yes	MP	Dead
P10	F/62	MDA5+DM-Act	Active/0.33	2	MDA5+++	33	7271	604	Yes	Yes	Yes	DX, TAC	Dead
P11[###]	F/51	MDA5+DM-Act	Active/0.38	3	MDA5+++	38	708.6	253	Yes	Yes	Yes	MP, TAC	Alive
P12	F/44	MDA5+DM-Act	Active/0.38	3	MDA5+++	29	230.6	266	Yes	Yes	Yes	MP	Alive
P13[#]	M/34	MDA5+DM-Rem	Remission/ 0.10	4	MDA5+++	24	428.5	345	Yes	No	Yes	MP, TAC	Alive
P14[##]	F/63	MDA5+DM-Rem	Remission/ 0.05	5	MDA5+++	60	127.3	319	Yes	No	Yes	MP, CTX	Alive
P15[###]	F/51	MDA5+DM-Rem	Remission/ 0.10	6	MDA5+++	14	252	256	Yes	No	Yes	MP, TAC	Alive
H01	F/42	HD	/	/	/	/	/	/	/	/	/	/	/

H02	F/37	HD	/	/	/	/	/	/	/	/	/	/	/	/
H03	M/44	HD.	/	/	/	/	/	/	/	/	/	/	/	/
H04	M/25	HD	/	/	/	/	/	/	/	/	/	/	/	/
PL1	F/54	MDA5+DM	Active/0.43	10	MDA5+++	220	1500	361	Yes	Yes	Yes	MP, TAC	Dead	

Note: Except PL1 was subject to lung scRNA-seq, others were used for peripheral T/B cell scRNA-seq.

Abbreviations: MSA, Myositis-specific autoantibodies; CK, creatine kinase; SF, serum ferritin; ILD, interstitial lung disease; MP, Methylprednisolone; CTX, cyclophosphamide; DX, dexamethasone; TAC, Tacrolimus; Pred, Prednisone; Aza, Azathioprine; MTX, methotrexate.

[#],[##],[###]: The same patients were indicated by the same symbols before and after treatment. The gray-scale values of MSA band were defined in the following manner: 11 to 25 units/L as +, 26 to 50 units/L as ++, and >50 units/L as +++.

Supplementary Table 2. Marker genes for cell clusters used in scRNA-seq (PBMC)

Main cell types	Cell subtypes	Reference markers
B cells	B Lineage	*CD79A/CD19*
	scB1-Tr	*CD9/SOX4/DUSP1*
	scB2-ISG	*ISG15/IFIT3/IFITM1*
	scB3-Naïve	*TCL1A/IL4R/IGHD*
	scB4-Unswitch Bm	*AIM2/EGR1/CD1C*
	scB5-Switch Bm	*AIM2/ITGB1/CRIP2*
	scB6-Atm	*AIM2/FGR/FCRL5*
	scB7-pASC	*XBP1/MZB1/MKI67/TOP2A*
	scB8-rASC	*XBP1/MZB1/IGHG1*
CD4+T cells	CD4T Lineage	*CD3D/CD4*
	scCD4T1-Naïve	*SELL/CCR7/LEF1*
	scCD4T2-ISG	*ISG15/IFIT3/IFITM1*
	scCD4T3-Tcm	*GPR183/MYADM/DUSP1*
	scCD4T4-Tfh	*CXCR5/DUSP5/KLRB1*
	scCD4T5-Th1	*CXCR3/CCL5/GZMK*
	scCD4T6-Th17	*CCR6/KLRB1/LGALS3*
	scCD4T7-Th2	*CXCR4/ITGB1/LGALS1*
	scCD4T8-Treg	*FOXP3/TIGIT/CTLA4*
	scCD4T9-Th22	*CCR4/CCR10/DUSP4*
CD8+T cells	CD8T Lineage	*CD3D/CD8*
	scCD8T1-Naïve	*SELL/CCR7/LEF1*
	scCD8T2-Tcm	*GPR183/IL7R/LTB*
	scCD8T3-GZMK+GZMB-Tm	*GZMA/GZMK/CMC1*
	scCD8T4-GZMK+ GZMB+ Tm	*GZMA/GZMK/GZMB*
	scCD8T5-GZMK- GZMB+ Tm	*GZMA/GZMB/FGFBP2*
	scCD8T6-ISG	*ISG15/IFIT3/IFITM1*
	scCD8T7-pTm	*MKI67/TOP2A/STMN1*
UN T cells	scgdT	*TRDC/TYROBP/TRDV2*
	scMAIT	*KLRB1/TBX21/PRF1*

Supplementary Table 3. Demographic data of recruited donors from an independent cohort for B cell validation (flow cytometry)

	HDs (n=16)	Ctrl IIM (n=20)	$MDA5^+$ DM (n=36)
Age, mean (range)	48.69(31, 63)	49.05 (23, 84)	51.19 (22, 69)
Sex (male/female)	37.5%(6/10)	25%(5/15)	27.8%(10/26)
Anti-MDA5 (%)	NA	0	36,100%*
Anti-Anti-Jo-1 (%)	NA	9 (45%)	0*
Anti-NXP2 (%)	NA	5 (25%)	0*
Anti-SAE (%)	NA	1 (5%)	0
Anti-TIF1r (%)	NA	1 (5%)	0
Anti-Mi-2 (%)	NA	3 (15%)	0
Anti-SRP (%)	NA	1 (5%)	0
CK (U/L), mean (range)	NA	1457 (20, 6148)	96.44* (14, 1048)
Ferritin (ng/ml), mean (range)	NA	479.2 (19.3, 1710)	1402* (65, 7027)
LDH(U/L), mean (range)	NA	440.1 (148, 758)	368.4* (169, 1687)
MITAX mean (range)	NA	0.40 (0.24,0.57)	0.36 (0.24,0.52)

Abbreviations: CK, creatine kinase; LDH, lactate dehydrogenase.
Note: Range, Minimum-Maximum;
*: Significant difference ($p<0.05$) between $MDA5^+$ DM and Ctrl IIM groups (two-tailed Mann-Whitney test).

Supplementary Table 4. Demographic data of recruited donors from an independent cohort for T cell validation (flow cytometry)

	HDs (n=16)	Ctrl IIM (n=16)	MDA5$^+$ DM (n=31)
Age, mean (range)	48.69(31, 63)	52.19 (23, 84)	50.32 (22, 69)
Sex (male/female)	37.5%(6/10)	31.25%(5/11)	29%(9/22)
Anti-MDA5 (%)	NA	0	31,100%*
Anti-Anti-Jo-1 (%)	NA	7 (43.75%)	0*
Anti-NXP2 (%)	NA	4 (25%)	0*
Anti-SAE (%)	NA	1 (6.25%)	0
Anti-TIF1r (%)	NA	1 (6.25%)	0
Anti-Mi-2 (%)	NA	2 (12.5%)	0
Anti-SRP (%)	NA	1 (6.25%)	0
CK (U/L), mean (range)	NA	1810 (27, 6148)	50.74* (14, 260)
Ferritin (ng/ml), mean (range)	NA	548.2 (104.2, 1710)	1258* (65, 7027)
LDH(U/L), mean (range)	NA	494.1 (228, 758)	376* (169, 1687)
MITAX, mean (range)	NA	0.39 (0.24,0.57)	0.35 (0.24,0.48)

Abbreviations: CK, creatine kinase; LDH, lactate dehydrogenase.
Note: Range, Minimum-Maximum;
*: Significant difference (p<0.05) between MDA5$^+$ DM and Ctrl IIM groups (two-tailed Mann-Whitney test).

Supplementary Table 5. Marker genes for cell clusters used in scRNA-seq (Lungs)

Main cell types	Cell clusters	Marker genes
Non-immune cells	Epithelial cells	*SPRR3/KRT13/CSTA*
	Alveolar type II cells	*SFTPB/SFTPC/SCGB3A2*
	Fibroblasts	*COL1A1/COL3A1/COL1A2*
	Endothelial cells	*CLDN5/ACKR1/EGFL7*
	Ciliated	*PIFO/C9orf24/CAPS*
Innate immune cells	Mast cells	*TPSAB1/TPSB2/CPA3*
	Macrophages	*S100A8/S100A9/CD68*
	DC	*CD1C/HLA−DPB1/CD74*
	NK cells	*TYROBP/NKG7/GZMB*
Adaptive immune cells	ISG- CD4 T cells	*CD3D/CD3E/CD4*
	ISG+ CD4 T cells	*CD3D/CD3E/CD4/ISG15/MX1*
	ISG- CD8 T cells	*CD3D/CD3E/CD8*
	ISG+ CD8 T cells	*CD3D/CD3E/CD8/ISG15/MX1*
	CD8 pT	*CD3D/CD3E/CD8/MKI67/TOP2A*
	B cells	*MS4A1/CD79A/IGHM*
	ASC	*MZB1/XBP1/IGHG1*

Supplementary Table 6. The clinical data at baseline between the CNI and CNI+TOFA groups

**Variables**	**CNI (n=22)**	**CNI+TOFA (n=14)**	***p***
Age(year)	55.5 (46.5， 60.5)	52 (43.5， 55.25)	0.2842
Gender(male/female)	9/13	4/10	0.5013
CK(U/L)	79 (28.25，60.25)	34 (26.5， 60.25)	0.1388
Ferritin(ng/ml)	1116 (625.8, 1705)	1040 (535， 1345)	0.5476
LDH(U/L)	359 (287.8,436)	293.5 (266.8, 391.8)	0.364
FVC(%)	66(51, 70)	64(55.5, 73.5)	0.7558
DLCO(%)	54(48, 67)	62(50, 70.5)	0.3802
Unable to perform PET, n(%)	7(31.82%)	4(28.57%)	1.0000
Non-survivor/survivor(n/n, %)	8/14(36.4%)	1/13(7.1%)	**0.0156**

Abbreviations: CNI, calcineurin inhibitor; CK, creatine kinase; DLCO, diffusing capacity of lung carbon monoxide; FVC, forced vital capacity; LDH, lactate dehydrogenase; TOFA, tofacitinib; PFT: Pulmonary Function Test.

Note: Continuous variables are expressed in medians and the 25th-75th percentiles of the interquartile range (IQR). Statistical significance was calculated by two-tailed Mann-Whitney test or Fisher’s test.

Supplementary Table 7. Reference genes used to calculate ISG and fibrosis scores

	ISG score		Fibrosis score
OAS3	*IFIT5*	*IFNGR2*	*ITGB1*
PLSCR1	*DDX58*	*WARS*	*SHH*
CMPK2	*IFIT3*	*TNFSF10*	*SMAD7*
MX2	*IFIT1*	*CCL3*	*CTNNB1*
STAT2	*RSAD2*	*FCGR1A*	*SIRT1*
PARP14	*DHX58*	*SERPING1*	*COL1A1*
GBP1	*SPATS2L*	*IFIT2*	*MMP2*
MT2A	*SAMD9*	*CCL5*	*NOX4*
OTOF	*PARP9*	*IFNG*	*TGFB1*
LY6E	*PARP12*	*CCL4*	*MMP14*
OASL	*IFI44L*	*GBP5*	*FN1*
HERC5	*EIF2AK2*	*IFITM1*	*CTGF*
MX1	*XAF1*	*APOL6*	*AKT1*
STAT1	*LGALS3BP*	*SOCS1*	*NOTCH1*
IFI44	*HERC6*	*ZC3HAV1*	*CCL2*
OAS2	*DDX60*	*GADD45B*	*KLF5*
IFIT1	*TMEM140*	*UNC93B1*	*PLAUR*
EPST11	*IFITM2*	*IFNLR1*	*PPARG*
IFI27	*AIF1*	*IRF7*	
ISG15	*HES4*	*IRF8*	
IFI6	*C1QA*	*PARP10*	
PSMB9	*DYNLT1*	*ISG20*	
PML	*IFNGR1*	*ZBP1*	
UBE2L6	*SCO2*	*HSH2D*	
NMI	*CASP1*	*IFNAR2*	
DRAP1	*CARD16*	*IFNAR1*	
BST2	*LGALS9*	*IP6K2*	
LAP3	*TCN2*	*REC8*	
FBXO6	*CXCL10*	*TAP1*	
IFI35	*IFITM3*	*SP100*	
OAS1	*IFNGR2*	*IRF9*	
SAMD9L	*WARS*	*CCL3*	
ADAR	*TNFSF10*	*FCGR1A*	
SP110	*CHMP5*	*IFI16*	
TRIM22	*PHF11*	*RBCK1*	
