# Supplementary material for: Single-cell profiling reveals distinct adaptive immune hallmarks in MDA5+ dermatomyositis with therapeutic implications
Source: Nat Commun. 2022 Oct 29;13:6458. doi: 10.1038/s41467-022-34145-4 (PMC9617246; doi:10.1038/s41467-022-34145-4)
Supplement: Supplementary file 3 — Reporting Summary [file 41467_2022_34145_MOESM3_ESM.pdf]

nature portfolio

Corresponding author(s): Xiaoming Zhang/Qiong Fu

Last updated by author(s): Sep 22, 2022

Reporting Summary

Nature Portfolio wishes to improve the reproducibility of the work that we publish. This form provides structure for consistency and transparency in reporting. For further information on Nature Portfolio policies, see our Editorial Policies and the Editorial Policy Checklist.

Statistics

For all statistical analyses, confirm that the following items are present in the figure legend, table legend, main text, or Methods section.

n/a	Confirmed	
☐	☒	The exact sample size (n) for each experimental group/condition, given as a discrete number and unit of measurement
☐	☒	A statement on whether measurements were taken from distinct samples or whether the same sample was measured repeatedly
☐	☒	The statistical test(s) used AND whether they are one- or two-sided<br>*Only common tests should be described solely by name; describe more complex techniques in the Methods section.*
☐	☒	A description of all covariates tested
☐	☒	A description of any assumptions or corrections, such as tests of normality and adjustment for multiple comparisons
☐	☒	A full description of the statistical parameters including central tendency (e.g. means) or other basic estimates (e.g. regression coefficient) AND variation (e.g. standard deviation) or associated estimates of uncertainty (e.g. confidence intervals)
☐	☒	For null hypothesis testing, the test statistic (e.g. F, t, r) with confidence intervals, effect sizes, degrees of freedom and P value noted<br>*Give P values as exact values whenever suitable.*
☒	☐	For Bayesian analysis, information on the choice of priors and Markov chain Monte Carlo settings
☒	☐	For hierarchical and complex designs, identification of the appropriate level for tests and full reporting of outcomes
☐	☒	Estimates of effect sizes (e.g. Cohen's d, Pearson's r), indicating how they were calculated

Our web collection on statistics for biologists contains articles on many of the points above.

Software and code

Policy information about availability of computer code

Data collection	For PBMC samples, the cell suspension of each sample was subjected to the Chromium Next GEM Single Cell 5′ Reagent Kit (V2) (10x Genomics, Pleasanton, CA) to prepare single cell 5′ gene expression libraries, 5′ cell surface protein libraries and V(D)J libraries following the manufacture's protocols (10× Genomics). For lung cell scRNA-seq, the Chromium Next GEM Single Cell 3′ Reagent Kit (V2) (10x Genomics, Pleasanton, CA) was used. The single cell libraries were sequenced on Illumina NovaSeq 6000 Systems using paired-end sequencing (150nt).
Data analysis	The scRNA-seq data were aligned and quantified by using Cell Ranger (v6.1.1) against the GRCh38 human reference genome. The preliminary counts were then used for downstream data analysis by Seurat (v4.0.2) including quality control filtering, data preprocessing, and data visualization. Then we used the DoubletFinder (v2.0.3) for doublets finding and Harmony (v0.1.0) for data intergrating.<br>We used TooManyCells (v2.2.0.0) to visualize the relationships in cell clades and the significant difference. Group preference of cell types was estimated by the STARTRAC-dist (v0.1.0) index. To annotate the function of different expression genes(DEGs), we used the ClusterProfiler (v3.19.0) and scMetabolism (V0.2.1) to perform the enrichment analysis in datasets org.Hs.eg.db (v3.12.0) and MSigDB (v7.4). To predict the potential transcriptional regulatory network, we performed the SCENIC analysis by using GRNboost2 (v0.1.6), AUCell (v0.99.5) and RcisTarget (v1.10.0). The relationship between the regulons with targets was visualized by Igraph (v1.3.0). To search for the interaction with lung fibrosis related cell subtypes, we performed the CellChat (v1.1.0) and CellPhoneDB (v2.0) to infer the ligand-receptor interaction.<br>All of BCR/TCR sequences were assembled and quantified following Cell Ranger vdj pipeline against GRCH38 reference genome. This pipeline used the standard IMGT reference database of human alleles. The TigGer (v1.0.0) was performed to infer novel alleles and subject-specific genotypes. SHazaM (v1.1.0) package was used to evaluate the somatic hypermutation (SHM) and selection strength. Finally, the pSTARTRAC-tran algorithm (v0.1.0) was used to calculate lineage tracking by clonotypes. The code of TCR/BCR diversity, clonality and jaccard index algorithm were publicly available on Github (https://github.com/Zechuan-Chen/scVDJplot).

For manuscripts utilizing custom algorithms or software that are central to the research but not yet described in published literature, software must be made available to editors and reviewers. We strongly encourage code deposition in a community repository (e.g. GitHub). See the Nature Portfolio guidelines for submitting code & software for further information.

Data

Policy information about availability of data

All manuscripts must include a data availability statement. This statement should provide the following information, where applicable:

- Accession codes, unique identifiers, or web links for publicly available datasets
- A description of any restrictions on data availability
- For clinical datasets or third party data, please ensure that the statement adheres to our policy

The single cell sequencing datasets of human healthy lungs used in this paper are publicly obtained from the Human Cell Atlas Data Coordination Platform/NCBI BIOPROJECT with the accession code PRJEB31843. The raw single-cell RNA sequencing data reported in this paper have been deposited at the National Genomics Data Center (https://ngdc.cncb.ac.cn) under the accession number HRA003082. The raw single-cell immune repertoire sequencing data have been deposited at the National Center for Biotechnology Information Sequence Read Archive (https://www.ncbi.nlm.nih.gov/sra) under the accession number PRJNA881644. The raw sequencing data are available for non-commercial purposes. Source data are provided with this paper. The remaining data are available within the Article or Supplementary Information.

Field-specific reporting

Please select the one below that is the best fit for your research. If you are not sure, read the appropriate sections before making your selection.

☒ Life sciences ☐ Behavioural & social sciences ☐ Ecological, evolutionary & environmental sciences

For a reference copy of the document with all sections, see nature.com/documents/nr-reporting-summary-flat.pdf

Life sciences study design

All studies must disclose on these points even when the disclosure is negative.

Sample size	No standard methods were used to predetermine sample size. We recruited patients who fulfilled 2017 EULAR/ACR Classification Criteria for Idiopathic Inflammatory Myopathies. The sample sizes are sufficient to provide stable single cell clustering results and to perform statistical analysis.
Data exclusions	For scRNA-Seq data, We excluded low quality cells through quality control pipeline, and cell types with less than 500 detected genes or more than 10% mitochondrial gene counts.
Replication	Findings derived from scRNA-seq analysis were validated by flow cytometry study performed in independent groups of MDA5+DM patients (Figures 2c, 3c, 7a and 7b) and all attempts were successful.
Randomization	No randomization was performed due to the nature of the study, as all patients were diagnosed with inflammatory myositis and treated by standard procedure.
Blinding	For scRNA-seq, flow cytometry and mIHC, the experimenters were blinded to the sample information. For analysis, the initial analysis for individual samples were also blinded to the analyzers; then for the group analysis, the sample information was notified to the analyzers, as the purpose was to identify the difference between groups.

Reporting for specific materials, systems and methods

We require information from authors about some types of materials, experimental systems and methods used in many studies. Here, indicate whether each material, system or method listed is relevant to your study. If you are not sure if a list item applies to your research, read the appropriate section before selecting a response.

Materials & experimental systems

n/a	Involved in the study	
☐	☒	Antibodies
☒	☐	Eukaryotic cell lines
☒	☐	Palaeontology and archaeology
☒	☐	Animals and other organisms
☐	☒	Human research participants
☒	☐	Clinical data
☒	☐	Dual use research of concern

Methods

n/a	Involved in the study	
☒	☐	ChIP-seq
☐	☒	Flow cytometry
☒	☐	MRI-based neuroimaging

Antibodies

Antibodies used	Information for flow cytometry antibodies is listed in “Methods-Flow cytometry”.

Antibodies used

1. FITC anti-human CD3 (Cat: 300406, Clone: UCHT1, Biolegend);
2. BUV395 anti-human CD3 (Cat: 564001, Clone: SK7, BD);
3. PerCPCy5.5 anti-mouse CD3 (Cat: 100218, Clone: 17A2, Biolegend);
4. APCCy7 anti-human CD4 (Cat: 357416, Clone: A161A1, Biolegend);
5. Alexa Fluor 700 anti-human CD8 (Cat: 300920, Clone: HIT8a, Biolegend);
6. BV570 anti-human CD14 (Cat: 301832, Clone: M5E2, Biolegend);
7. PE anti-human CD19 (Cat: 302254, Clone: HIB19, Biolegend);
8. BV711 anti-human CD19 (Cat: 302246, Clone: HIB19, Biolegend);
9. BV785 anti-human CD19 (Cat: 302240, Clone: HIB19, Biolegend);
10. BV711 anti-human CD20 (Cat: 302342, Clone: 2H7, Biolegend);
11. PECy7 anti-human CD27 (Cat: 302838, Clone: O323, Biolegend);
12. BV650 anti-human CD27 (Cat: 302828, Clone: O323, Biolegend);
13. APC anti-human CD38 (Cat: 303510, Clone: HIT2, Biolegend);
14. BV785 anti-human CD45RA (Cat: 304140, Clone: HI100, Biolegend);
15. Biotin anti-human TCRgd (Cat: 331206, Clone: B1, Biolegend);
16. Alexa Fluor 488 anti-human MX1 (Cat: ab237298, Clone: EPR19967, Abcam);
17. PE anti-human ISG15 (Cat: IC8044P, Clone: 851701, R&D);
18. PECy5.5 anti-human Ki67 (Cat: 35-5699-42, Clone: 20Raj1, eBioscience);
19. Streptavidin BV570 (Cat: 405227, Biolegend).

Information for immunohistochemistry antibodies is listed in “Methods- Multiplex immunohistochemistry”.

20. anti-human CD8 (Cat: M7103, Clone: C8/144B, Dako);
21. anti-human Ki-67 (Cat: ab16667, Clone: SP6, Abcam);
22. anti-human MX1(Cat: 13750-1-AP, Clone: Rabbit polyclonal IgG, Proteintech).
23. ImmPRESS HRP Goat Anti-Rabbit IgG Polymer Detection Kit (Cat: MP-7451-50, Vectorlabs)
24. ImmPRESS HRP Goat Anti-Mouse IgG Polymer Detection Kit (Cat: MP-7452-50, Vectorlabs)

Validation

All antibodies used are commercially available and their manufacturers provided their validation documents. They have been validated for flow cytometry and/or IHC staining.

1.https://www.biolegend.com/en-us/products/fitc-anti-human-cd3-antibody-863
2. https://www.bdbiosciences.com/en-us/search-results?searchKey=564001
3.https://www.biolegend.com/en-us/products/percp-cyanine5-5-anti-mouse-cd3-antibody-5596
4.https://www.biolegend.com/en-us/products/apc-cyanine7-anti-human-cd4-antibody-13182
5.https://www.biolegend.com/en-us/products/alexa-fluor-700-anti-human-cd8a-antibody-3434
6.https://www.biolegend.com/en-us/products/brilliant-violet-570-anti-human-cd14-antibody-7366
7.https://www.biolegend.com/en-us/products/pe-anti-human-cd19-antibody-719
8.https://www.biolegend.com/en-us/products/brilliant-violet-711-anti-human-cd19-antibody-8519
9.https://www.biolegend.com/en-us/products/brilliant-violet-785-anti-human-cd19-antibody-7967
10.https://www.biolegend.com/en-us/products/brilliant-violet-711-anti-human-cd20-antibody-8926
11.https://www.biolegend.com/en-us/products/pe-cyanine7-anti-human-cd27-antibody-8434
12.https://www.biolegend.com/en-us/products/brilliant-violet-650-anti-human-cd27-antibody-7660
13.https://www.biolegend.com/en-us/products/apc-anti-human-cd38-antibody-744
14.https://www.biolegend.com/en-us/products/brilliant-violet-785-anti-human-cd45ra-antibody-7972
15.https://www.biolegend.com/en-us/products/biotin-anti-human-tcr-gamma-delta-antibody-4551
16.https://www.abcam.com/alexa-fluor-488-mx1-antibody-epr19967-ab237298.html
17.https://www.rndsystems.com/cn/products/human-isg15-ucrp-pe-conjugated-antibody-851701_ic8044p
18.https://www.thermofisher.cn/cn/zh/antibody/product/Ki-67-Antibody-clone-20Raj1-Monoclonal/35-5699-42
19.https://www.biolegend.com/en-us/products/brilliant-violet-570-streptavidin-7458
20.https://www.agilent.com/cs/library/packageinsert/public/SSM7103CEEFG_01.pdf
21. https://www.abcam.com/ki67-antibody-sp6-ab16667.html
22. https://www.ptglab.com/Products/MX1-Antibody-13750-1-AP.htm
23. https://www.vectorlabs.com/products/enzyme-polymer/immpress-hrp-goat-anti-rabbit-igg-kit
24. https://www.vectorlabs.com/products/enzyme-polymer/immpress-hrp-goat-anti-mouse-igg-kit

Human research participants

Policy information about studies involving human research participants

Population characteristics

All the MDA5+DM patients in the study were Chinese Han nationality. Patients' demographic information as well as laboratory data, clinical manifestations and treatments were recorded and presented in Table S1, S3, S4 and S6.

Recruitment

All IIM patients (Ctrl IIM and MDA5+ DM) were recruited consecutively from Renji Hospital as they fulfilled the following criteria the 2017 EULAR/ACR Classification Criteria for Idiopathic Inflammatory Myopathies. Peripheral blood samples were obtained during the same clinical visit after patient's signed agreement. Samples were immediately processed or frozen in Renji Rheumatology biobank. We are not aware of any specific bias which could affect the recruitment of patients.

Ethics oversight

This study was reviewed and approved by the Institutional Review Board of Renji Hospital (ID: 2013-126), Shanghai, China. Informed consent was obtained from all study participants. All studies were performed in accordance with the Declaration of Helsinki.

Note that full information on the approval of the study protocol must also be provided in the manuscript.

Flow Cytometry

Plots

Confirm that:

☒ The axis labels state the marker and fluorochrome used (e.g. CD4-FITC).

☒ The axis scales are clearly visible. Include numbers along axes only for bottom left plot of group (a 'group' is an analysis of identical markers).

☒ All plots are contour plots with outliers or pseudocolor plots.

☒ A numerical value for number of cells or percentage (with statistics) is provided.

Methodology

Sample preparation	Human peripheral blood mononuclear cells (PBMCs) were isolated by Lymphoprep (Axis-Shield) density gradient centrifugation.PBMCs were first stained with Zombie Yellow (Cat: 423104, Biolegend) to remove dead cells. For surface staining, cells were stained with fluorochrome-labelled antibodies in the staining buffer (PBS with 5% FBS, 2mM EDTA and 0.09% NaN3) at room temperature for 15min. For intracellular staining, cells were fixed and permeabilized by a Cytofix/ Cytoperm Fixation/Permeablization Kit (Cat: 554714, BD) and intracellularly stained with appropriated fluorochrome-labelled antibodies in the Permeablization buffer for 30 minutes at 4℃. Stained cells were washed twice in the staining buffer and filtered with 70μm cell-strainer nylon mesh (Falcon) before doing the acquisition.
Instrument	For cell sorting: FACSAria II (BD); for cell anlaysis: LSRFortessa, 5-laser (BD)
Software	Flowjo Version 10.7.1 (BD)
Cell population abundance	We sorted CD19+ B cells and CD3+ T cells from PBMCs to perform scRNA-seq. The purities of the sorted B and T cells were routinely more than 98%, rechecked by flow cytometry.
Gating strategy	The lymphocytes were first gated within the lymphocyte region in the FSC/SSC plots, then the doublets were removed by gating the diagonal cells in the FSC-H vs FSC-A plots. Dead cells were removed by gating Zombie Yellow-negative cells. The specific cell populations were gated by their unique markers, such as CD3 for T cells and CD19 for B cells. The detailed gating strategies were provided in Figure S1a, S2c, S3e, and S7a.

☒ Tick this box to confirm that a figure exemplifying the gating strategy is provided in the Supplementary Information.
